# Reverse Shoulder Arthroplasty versus Non-Operative Treatment of Three-Part and Four-Part Proximal Humerus Fractures in the Elderly Patient: A Pooled Analysis and Systematic Review

**DOI:** 10.3390/jcm13113344

**Published:** 2024-06-06

**Authors:** Thomas P. Bosch, Frank J. P. Beeres, Steven Ferree, Inger B. Schipper, Roland S. Camenzind, Ruben J. Hoepelman, Björn-Christian Link, Ingmar F. Rompen, Reto Babst, Bryan J. M. van de Wall

**Affiliations:** 1 Department of Health Sciences and Medicine, University of Lucerne, 6002 Luzern, Switzerland; 2 Department of Trauma Surgery, Leiden University Medical Centre, 2300 RC Leiden, The Netherlands; 3 Department of Orthopedics and Trauma Surgery, Lucerne Cantonal Hospital, 6000 Luzern, Switzerland; 4 Department of Surgery, University Medical Center Utrecht, 3584 CX Utrecht, The Netherlandsr.j.hoepelman-2@umcutrecht.nl (R.J.H.); 5 Department of Surgery, University Hospital Heidelberg, 69117 Heidelberg, Germany

**Keywords:** arthroplasty complications, complex humerus fracture, frail trauma patient, non-operative treatment, reverse shoulder arthroplasty

## Abstract

**Background**: The treatment of complex proximal humerus fractures in elderly patients is not yet fully elucidated. Of all treatment options, reverse shoulder arthroplasty (RSA) and non-operative treatment (NOT) appear to provide the best results. Evidence to guide the choice between the two is sparse. Therefore, this review provides an overview of the available evidence on RSA versus NOT. **Methods**: Studies comparing complex proximal humerus fractures in patients aged >65 years treated either with RSA or NOT were included for systematic review and direct comparison via pooled analysis of patient-rated outcome and range of motion. Indirect comparison of case series and non-comparative studies on either treatment was performed separately. **Results**: Three comparative studies including 77 patients treated with RSA and 81 treated non-operatively were analysed. The RSA group scored better for both the Constant–Murley score (mean difference 6 points) and DASH score (mean difference 8 points). No differences were detected in ASES, PENN score, pain scores, or range of motion between treatment groups. The most common complications for RSA were infection (3%), nerve injury (2%), and dislocation (2%). Reoperation was required in 5%. In the NOT group, common complications included malunion (42%), osteonecrosis (25%), and non-union (3%); no reoperation was required. Patient satisfaction was equal in both groups. **Conclusions**: The functional outcomes and range of motion after RSA seemed satisfactory and potentially superior to NOT in elderly patients. Patient satisfaction was comparable despite a high malunion and osteonecrosis rate in the non-operative treatment group, which did not require re-interventions.

## 1. Introduction

Proximal humerus fractures (PHFs) are common injuries and account for 5% of all adult fractures [1]. They represent one of the most common fractures in elderly patients with poor bone quality. Due to an aging population, their incidence is expected to rise in the upcoming decades, increasing costs and burden on an already stretched healthcare system. Optimising the treatment of these fractures is vital to keeping our healthcare system sustainable.

Treatment options in elderly patients with PHF generally consist of non-operative treatment (NOT), open reduction and internal fixation (ORIF), hemiarthroplasty (HA), and reverse total shoulder arthroplasty (RSA). Frequently, the Neer classification is used to guide treatment decisions. The Neer classification categorises PHFs based on the number and displacement of fracture fragments [2]. Simple PHFs are generally treated non-operatively with satisfactory results. With increasing complexity of the fracture pattern, outcomes are thought to be poorer when left untouched. As a result, surgeons frequently revert to one of the aforementioned surgical treatment options to counteract this inverse relation. The advantage of ORIF is that the native joint is preserved, generally offering better functional results than arthroplasty. However, elderly patients are at a considerable risk of developing humeral head necrosis, potentially necessitating arthroplasty when symptomatic. Offering primary arthroplasty for fractures has the benefit of preventing secondary surgery related to failure of ORIF. However, when complications such as infection occur, they may have disastrous consequences for patients [3].

The debate on the optimal treatment modality in elderly patients with three- or four-part PHF is still ongoing. Several randomized studies and meta-analysis found no added benefit of surgical management with ORIF or HA compared to NOT [4,5]. However, studies did find better outcomes for patients treated with RSA compared with ORIF or HA [6,7,8].

RSA has become an increasingly popular surgical modality for treating three- and four-part proximal humerus fractures [9]. Its superior results in comparison to other surgical options in recent studies could be an explanation for its growing use. However, in elderly patients, range of motion and functional outcome after NOT appear to be acceptable as well, even in displaced fractures [4,5,10,11]. To date, there have been no systematic reviews comparing the results of NOT with those of RSA for treatment of three- and four-part proximal humerus fractures.

The goal of this systematic review is to compare the patient-reported functional outcome and range of motion of RSA compared with NOT in elderly patients who sustained three- or four-part proximal humerus fractures.

## 2. Methods

This study was set up as a systematic review to be performed according to the Preferred Reporting Items for Systematic Reviews and Meta-analysis (PRISMA) checklist [12] and Meta-analysis of Observational Studies in Epidemiology (MOOSE) [13]. No protocol has been published for this study.

### 2.1. Search Strategy and Selection Criteria

Databases of PubMed, Embase, CINAHL, and CENTRAL were searched on 18 January 2023 for studies on complex proximal humerus fractures in patients aged 65 years and older comparing RSA to NOT (Supplementary Material Table S1). Two reviewers screened titles and abstracts independently for eligibility. All studies comparing RSA and NOT were included for systematic review and (if possible) direct comparison via pooled analysis. Indirect comparison via systematic review of case series and non-comparative studies on either of the two treatment modalities was performed separately.

Full-text screening was performed by the same authors. Inclusion criteria were comparative studies on RSA versus NOT for treatment of complex proximal humeral fractures (classified as Neer three- or four-part displaced or AO (Arbeitsgemeinschaft für Osteosynthesefragen) classification 11-B or 11-C), age over 65 years (when not specified in the inclusion criteria, then mean age of 70 or older in the baseline characteristics), functional outcome and/or range of motion reported with a minimal follow up of 12 months. Non-operative treatment was defined as no invasive intervention performed (thus excluding open and closed reduction with or without Kirschner wire fixation). Exclusion criteria were congress or meeting abstracts, biomechanical, animal, or cadaver studies, no English, Dutch, or German full text available. Disagreements on study eligibility and quality were discussed and the decision to perform meta-analysis was reached through consensus of the authors. Reviews of the references of included studies were performed to identify studies missed in the initial search.

### 2.2. Data Extraction

One author extracted the data using a prespecified extraction sheet, includingyear of publication, study design, time period of study, follow up, patient characteristics, fracture classification and distribution, prosthesis type, cemented yes/no, approach used, reinsertion of tuberosities yes/no, outcome scores, pain score, and satisfaction with treatment modality. Regarding complications, the following were extracted from the studies: radiographic outcomes including loosening of stem or baseplate, scapular notching, and anatomic tuberosity healing for the RSA group and nonunion, malunion, and osteonecrosis for the NOT group. Furthermore, infection, dislocation, iatrogenic fracture, and revision surgery were noted.

### 2.3. Quality Assessment

The included studies were assessed by one author using the Methodological Index for Non-Randomized Studies (MINORS) tool. This validated tool for assessing methodological quality of studies rates studies from scores 0 to 24 (Supplementary Material Table S2). Pooled analysis was performed only when the following criteria were met: equivalence of baseline characteristics in the two treatment groups and availability of a minimum of three studies reporting on the outcome(s) of interest.

### 2.4. Study Outcome

The primary outcomes were functional outcome scores and range of motion after at least 12 months. Secondary outcomes were all complications, pain, and satisfaction with treatment.

### 2.5. Statistical Analysis

Variables were presented as per the original studies. Weighted average and mean difference of outcome scores were calculated when more than two studies reported on the same scale or questionnaire. The pooled mean scores were calculated through weighting of study size. Excel was used for data collection, calculation of pooling results, and analysis.

## 3. Results

**Figure 1:**
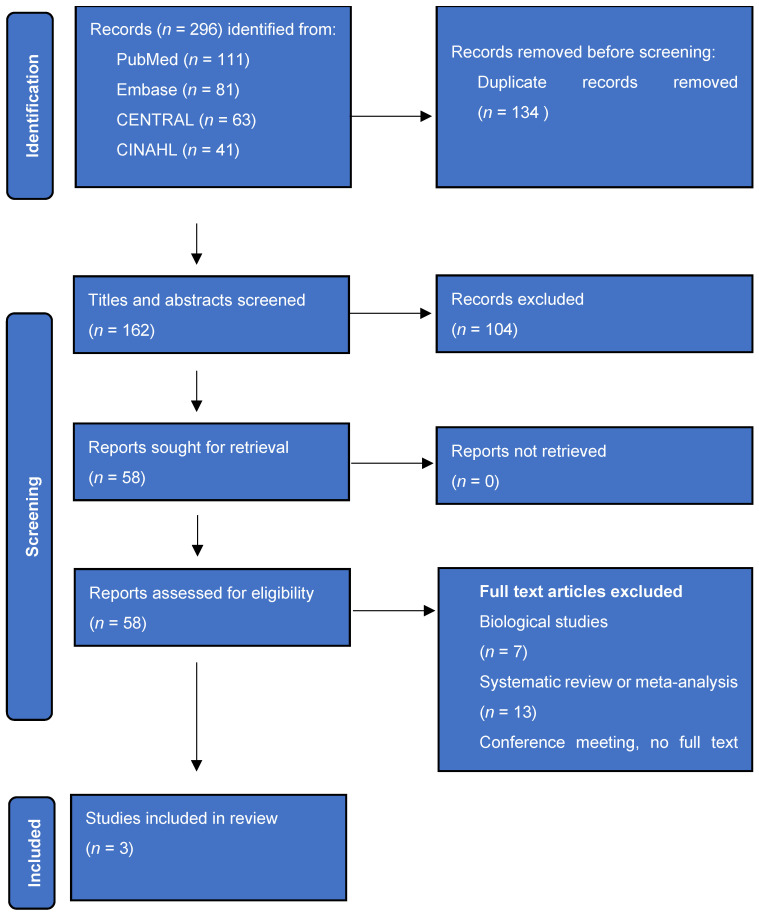
Flow diagram of search and selection of comparative studies.

The flowchart of the literature search and comparative study selection is shown in Figure 1. Three studies were included; one randomized clinical trial (Lopiz et al.) and two retrospective cohort studies (Chivot et al. and Roberson et al.) [14,15,16].

### 3.1. Comparative Studies

#### 3.1.1. Study Characteristics and Quality Assessment

The three studies included 158 patients; 77 underwent RSA and 81 had NOT. Table 1 provides the baseline characteristics of the studies. Lopiz et al. [14] and Roberson et al. [16] both included more (87% and 75% respectively) four-part fractures than Chivot et al. [15] Roberson et al. [16] included patients in the NOT group who were offered RSA as indicated by the surgeon but declined. The percentage lost to follow-up was 5% in the study by Lopiz et al. [14], 50% for Chivot et al. [15], and Roberson et al. did not provide data on eligible numbers of patients or follow-up. Table 2 shows the MINORS scores ranging from 13 to 24. In all included studies, the deltopectoral approach was used for the placement of the RSA.

#### 3.1.2. Functional Outcomes

Two studies [14,15] reported on the Constant–Murley score (CMS). The scores appeared to be significantly better in the RSA group in both studies, with a mean difference of six points (Table 3). Two studies [14,15] reported on the Disability of the Arm, Shoulder and Hand Questionnaire (DASH) score. The randomized clinical trial found a significantly better score in the RSA group, with a difference of eight points. One observational study found approximately the same difference in favour of RSA, but this was not statistically significant. No differences were found in the American Shoulder and Elbow Surgeons shoulder score (ASES) or Penn shoulder score (PENN) in the two studies that reported on these outcomes.

#### 3.1.3. Range of Motion

Chivot et al. [15] found better ROM in the RSA group for all movements. Lopiz et al. [14] found better anterior elevation in the RSA group compared with NOT but not better external or internal rotation. Abduction was investigated only by Lopiz et al. [14] and they found no difference (abduction >90 degree; RSA 72% versus NOT 42%, p0.064). Roberson found no improvements in anterior elevation or external rotation (Table 4).

#### 3.1.4. Complications

An overview of the complications for both RSA and NOT in the three included comparative studies is given in Table 5. In total there were four (5%) reoperations reported in the three studies; two for infection (one debridement and implant retention procedure and one removal of prosthesis), one for dislocation of the RSA, and one for postoperative stiffness.

#### 3.1.5. Pain and Satisfaction

Only Lopiz et al. [14] reported less pain in the RSA group, with VAS 0.9 versus 1.6 in the NOT group (*p* = 0.011). Roberson and Chivot et al. found no differences in pain scores [15,16].

With regard to treatment satisfaction, Lopiz et al. [14] reported that 100% of the RSA group versus 93% in NOT ‘would undergo the same treatment again based on the achieved result’ (*p* = 0.24). Chivot et al. also found higher satisfaction in the RSA group, with 93% versus 84% in the NOT group (*p* = 0.03).

#### 3.1.6. Case Series and Non-Comparative Studies on RSA and NOT

A total of 29 studies (1550 patients) reporting on RSA in case series or comparing elderly patients treated for complex proximal humeral fractures with treatments other than NOT were included for review of functionality and range-of-motion outcomes and complications [7,8,17,18,19,20,21,22,23,24,25,26,27,28,29,30,31,32,33,34,35,36,37,38,39,40,41,42,43]. Five case series and non-comparative studies after NOT were included (249 patients) (Supplementary Materials Tables S4 and S6) [10,44,45,46,47]. Weighted mean functional outcome scores after RSA were CMS 60, DASH 25, ASES 76, OSS 39, and Simple Shoulder Test 7.8 (Supplementary Materials Table S5). For NOT, the weighted mean scores were CMS 60, DASH 33.0, and OSS 36 (Supplementary Materials Table S6). Range of motion in degrees after RSA was anterior elevation 122, abduction 112, and external rotation 22 (Supplementary Materials Table S5). Twenty-five studies reported complications after RSA. The average rate of complications was 4.6% (infection 2%, dislocation 1.6%, and iatrogenic fracture 1%). The revision rate for infection, dislocation, iatrogenic fracture, or aseptic loosening in all studies averaged 4%.

## 4. Discussion

This review describes the differences in functional outcomes after RSA versus NOT in elderly patients with complex proximal humerus fractures. Functional outcomes and range of motion after RSA seemed better compared with NOT in comparative studies. The rate of complications after RSA was low at 3%, with an overall revision rate of 5%.

So, what are the clinical implications following these findings? It is likely that surgeons will have treated-higher demand patients with more complex fractures with RSA and lower-demand, less complex fractures non-operatively. This assumption is based on the fact that multiple case series have mentioned this in relation to their patient selection for RSA [7,8,20,23,33]. Although only discussed in one of the included observational comparative studies, it is likely that this was also the case here [16]. The big question remains: to what extent are the better results found in the RSA group attributable to the treatment itself and not to the type of patient (high-demand) or the complexity of the fracture? The one RCT included in this review that could have shed some light on this matter did not find any statistical significant difference in functional outcomes; however, scores for all outcomes were consistently better in the RSA group. On top of that, the large quantity of non-comparative studies point in the same direction, so it may be safe to say that RSA is likely to have some beneficial effect on patient outcomes. The magnitude of this effect remains unclear and should be further investigated.

Nevertheless, the results of this review still are valuable; they contribute to the knowledge on specific aspects of this topic. Assuming a selection bias to be present, the results shows that if we continue to offer RSA to high-demand patients with complex fractures and non-operative treatment to the lower-demand patients with less complex fractures, we can achieve satisfactory results in our day-to-day practice. It would be interesting to see future studies taking this confounding factor out of the equation, to truly understand how both treatment modalities behave in both the high-demand/complex fracture and low-demand/low complex fracture patient population.

A pragmatic approach in the treatment of elderly patients with complex proximal humerus fractures could be to consider RSA in cases where NOT has led to unfavourable outcomes. Several studies have shown that delayed RSA (>30 days) provides equal functional outcomes and range of motion to early RSA (<30 days) [16,48,49,50]. Interestingly, patients treated with RSA for sequalae of NOT potentially outperform patients after NOT on both functionality and range-of-motion scores [51]. In other words, conservative management and offering RSA either directly or in cases of failed conservative treatment, are both viable strategies for treating complex proximal humerus fractures in the elderly.

Non-anatomic healing and resorption of the greater tuberosity is frequently seen in patients who undergo RSA. The clinical relevance of this occurrence remains questionable. Several studies found that it was related to poor functional outcomes and range of motion. However, an equal number of studies did not find such a relation [24]. Therefore, it remains unclear whether non-anatomic healing and resorption should be regarded as complications or not. The same applies for malunion and osteonecrosis in the NOT group. These are regarded as complications; however, their relevance is questionable. Many patients with osteonecrosis are asymptomatic and do not require intervention [52]. Moreover, malunion is practically assured when complex fractures are treated conservatively. This is also reflected in the results of the present study; despite the high incidence of malunion and osteonecrosis, reintervention rates were low in the NOT group. It should, however, be acknowledged that it remains unclear whether a deliberate choice to refrain from revision surgery was made (for example because of severe comorbidities) or patients were truly satisfied with the results.

With regard to risk of other complications (infection, dislocation, nerve injury, reintervention) with RSA, it should be kept in mind that although complication and revision rates are low and long-term follow-up of RSA shows high arthroplasty survival rates and good clinical results, these results deteriorate over time [53]. Complication rates also appear to increase over time [54]. Furthermore, as previously stated, revision rate is not the same as success rate. An elderly patient with complications after initial RSA might not consent to revision surgery and might accept a less favourable result than would have been achieved with NOT. It is thus unclear whether the advantage of RSA over NOT is sustained over time and whether it is worth the risk of complications.

The burden on the healthcare system due to fractures has grown exponentially in recent decades. This is mainly attributed to a continuously expanding and aging population with higher expectations in the context of an increasingly demanding society [1,55]. Inevitably, the increased burden poses higher costs, putting the sustainability of countries’ healthcare systems at risk [56,57]. As PHFs are the second most common fractures in elderly people after hip fractures, it is important to consider the costs associated with the treatment of complex PHFs. The initial costs of RSA are higher than NOT, as expected, because of the cost associated with facilitating an RSA operation and the duration of the hospital stay. Yet, these costs seem to pay out over time. In 2022, Abdel Khalik et al. conducted a cost–utility analysis comparing HA, ORIF, RSA, and NOT in elderly patients, aged 75 years and older, with complex proximal humerus fractures. The study determined the reported inpatient cost QALY ratio in NOT and RSA to be 2584 CAD/QALY and 3077 CAD/QALY, respectively. Despite these higher upfront costs, RSA was deemed a more cost-effective treatment strategy for complex proximal humerus fractures in patients older than 65 years compared with NOT, because of its superiority in functional and clinical outcomes over time even in the older population [58].

## 5. Limitations

This review highlights the advantages of RSA compared with NOT. To our knowledge, this is the first review to address outcomes after RSA versus NOT for complex proximal humerus fractures specifically in elderly patients. Unfortunately, no meta-analysis was possible due to the small number of studies comparing only three-part and four-part proximal humerus fractures and heterogeneity in the study population (age and fracture type). Another limitation of our review is the lack of non-comparative studies reporting on NOT outcomes to put the results in perspective, like the extensive data on RSA outcomes. Finally, as previously stated, it is likely that selection bias might have occurred in the included observational studies and case series.

## 6. Conclusions

The functional outcomes and range of motion after RSA seem satisfactory and potentially superior to those achieved with NOT in elderly patients. The complication rate was acceptably low and an overall revision rate of 5% was found. These results should, however, be viewed in light of distinct differences in patient characteristics between treatment groups.

Which geriatric patients with complex proximal humerus fractures benefit from RSA should be the focus of future research. The NITEP (https://pubmed.ncbi.nlm.nih.gov/30700485/, accessed on 16 April 2024) and ReShAPE trials (https://pubmed.ncbi.nlm.nih.gov/30700485/, accessed on 16 April 2024) might shed some light on this topic and guide future treatment choices.

## 7. Future Directions

To further explore which elderly PHF patients most benefit from NOT and which from RSA without potential selection bias, a randomized controlled trial comparing treatment modalities including an adequate sample size and follow-up time is necessary. Furthermore, future studies should incorporate comprehensive assessments of both clinical and radiographic outcomes, including functional recovery, pain relief, range of motion, fracture healing, and implant positioning. Follow-up should extend beyond one year to evaluate the durability of treatment effects and the occurrence of late complications. Additionally, subgroup analyses based on patient characteristics and fracture patterns should be conducted to identify factors predictive of treatment success and guide physicians in personalised treatment decisions. Ultimately, the findings from future studies will contribute to facilitating evidence-based guidelines and improving the management of complex proximal humerus fractures in the elderly, enhancing patient outcomes and quality of life.

## Figures and Tables

**Table 1:** Baseline characteristics of studies after reverse total shoulder arthroplasty versus non-operative treatment.

Author	Design	Number of Patients		Age Mean in Years (Sd)		% Female		% Neer 4-part		Tuberculum Management	Prothesis Used	Follow-Up Months
		RSA	NOT	RSA	NOT	RSA	NOT	RSA	NOT			Average
Lopiz	RCT	29	30	82 (3.4)	85 (4.8)	86%	87%	87%	83%	Reattachment	Delta SMR	12
Chivot	Retro	28	32	77 (70–92)	79 (70–92)	78.6%	93.8%	35.7%	25%	Reattachment	Zimmer	32
Roberson	Retro	20	19	71 (NR)	71 (NR)	95%	78.9%	75%	21%	NR	DJO Zimmer	29/53 ^
		77	81									

Retro: retrospective study, RCT: randomized clinical trial, RSA: reversed total shoulder arthroplasty, NOT: non-operative treatment, NR: not reported, Delta: Delta XTEND Reverse Shoulder System prosthesis (DePuy, Warsaw, IN, USA), SMR: SMR Modular Shoulder System (Systema Multiplana Randelli; Lima-LTO, San Daniele del Friuli, Italy), Zimmer: The Trabecular Metal Reverse Shoulder System implant (Zimmer Biomet, Warsaw, IN, USA), DJO: Reverse Shoulder Prosthesis (DJO Surgical, Austin, TX, USA), ^: follow-up NOT group 29 months, RSA group 53 months.

**Table 2:** Quality assessment for studies after reverse total shoulder arthroplasty versus non-operative treatment.

	Randomized Clinical Trials	Lopiz 2019 [14]	Retrospective Studies	Chivot 2018 [15]	Roberson 2017 [16]
Clearly stated aim		2		2	2
Inclusion of consecutive patients		2		2	1
Prospective data collection		2		1	0
Appropriate endpoints		2		2	1
Unbiased assessment endpoints		2		0	0
Appropriate follow-up (>1 year)		2		2	2
Loss to follow-up <5%		2		1	0
Prospective calculation study size		2		0	0
Adequate control group		2		2	2
Contemporary groups		2		2	2
Baseline quivalence of groups		2		2	2
Adequate statistical analysis		2		2	1
Total score		24		18	13

**Table 3:** Functional outcome scores after reverse total shoulder arthroplasty versus non-operative treatment.

Functional Outcome Score	Study	RSA	NOT	*p*-Value
CMS *	Lopiz et al. [14]	61.7	55.7	0.07
	Chivot et al. [15]	56.5	50.5	**0.03 ^**
DASH **	Lopiz et al. [14]	20.7	28.8	0.08
	Chivot et al. [15]	38.7	31.2	0.11
PENN *	Roberson et al. [16]	70	73	0.7
ASES *	Roberson et al. [16]	72	72	0.99

CMS: Constant–Murley Score, DASH: Disability of the Arm, Shoulder and Hand questionnaire, ASES: American Shoulder and Elbow Surgeons shoulder score, PENN: Penn shoulder score, RSA: reverse shoulder arthroplasty, NOT: non-operative treatment, *: ranging 0–100 with higher score indicating better function, **: ranging 0–100 with lower score indicating better function, ^: difference in favour of RSA.

**Table 4:** Range-of-motion scores after reverse total shoulder arthroplasty versus non-operative treatment.

Range of Motion	Study	RSA	NOT	*p*-Value
Forward flexion	Lopiz et al. [14]	133	115	**0.028 ^**
	Chivot et al. [15]	110	98	**0.0005 ^**
	Roberson et al. [16]	119	120	0.87
	Weighted average	120.8	109.8	
External rotation	Lopiz et al. * [14]	5.2	4.4	0.293
	Chivot et al. [15]	19	9	**0.0002 ^**
	Roberson et al. [16]	31	23	0.06
Internal rotation **	Lopiz et al. [14]	65% (41/24)	48% (34/14)	0.211
	Chivot et al. [15]	46% (17/28)	22% (9/13)	**0.04 ^**

RSA: reverse shoulder arthroplasty, NOT: non-operative treatment, *: range of motion part of Constant–Murley Score, **: percentage patients with internal rotation above sacro-iliac joint level, (% lumbar/% thoracic), ^: difference in favour of RSA.

**Table 5:** Complications in included studies after RSA and NOT for proximal humerus fracture in elderly patients.

	RSA	NOT
N	77	81
RSA complications		
Dislocation	2 (2.6%)	
Nerve injury	2 (2.6%)	
Infection	2 (2.6%)	
Iatrogenic fracture	0	
Anatomic greater tuberosity healing	40 (52%)	
Greater tuberosity non-anatomic healing or resorption	17 (22%)	
Scapular notching	0	
Baseplate loosening	0	
Humeral stem loosening	0	
Revision or subsequent operation	4 (5.2%)	0
NOT complications		
Osteonecrosis		20 (24.7%)
Malunion		34 (42%)
Nonunion		2 (2.5%)

N: number, RSA: reverse shoulder arthroplasty, NOT: non-operative treatment.

## Supplementary Materials

The following supporting information can be downloaded at https://www.mdpi.com/article/10.3390/jcm13113344/s1: Table S1: Syntax of literature search on reverse total shoulder arthroplasty compared with non-operative treatment for proximal humeral fractures in the elderly; Table S2: Quality assessment according to the MINORS criteria; Table S3: Baseline characteristics of non-comparative studies and case series on RSA; Table S4: Functional outcome scores and weighted mean scores of case series and non-comparative studies on RSA; Table S5: Range of motion and weighted mean scores of case series and non-comparative studies on RSA; Table S6: Functional outcome scores and weighted mean scores of NOT cohorts from studies not compared with RSA.
