# Supplementary material for: Reverse Shoulder Arthroplasty versus Non-Operative Treatment of Three-Part and Four-Part Proximal Humerus Fractures in the Elderly Patient: A Pooled Analysis and Systematic Review
Source: J Clin Med. 2024 Jun 6;13(11):3344. doi: 10.3390/jcm13113344 (PMC11172441; doi:10.3390/jcm13113344)
Supplement: Supplementary file 1 [file jcm-13-03344-s001.zip › jcm-2994209-supplementary.pdf]

Supplementary Material Table S1. Syntax of literature search on reverse total shoulder arthroplasty compared to not operative treatment for proximal humeral fractures in the elderly

Database	Syntax
Pubmed/MEDLINE (n=111)	(("Conservative Treatment"[Mesh] OR "non operative" OR "non surgical" OR nonoperative OR nonsurgical OR conservative OR sling) AND ("Arthroplasty, Replacement, Shoulder"[Mesh] OR Hemiartrop* OR "Reverse shoulder arthrop*" OR "Shoulder Arthrop*")) AND ("Shoulder Fractures"[Mesh] OR "proximal humerus fracture" OR "proximal humeral fracture" OR "shoulder fracture")
Embase (n=81)	(conservative treatment:ti,ab OR non operative:ab,ti OR non surgical:ti,ab OR sling:ti,ab OR nonoperative:ti,ab) AND (arthroplasty:ti,ab OR reverse shoulder arthrop:ab,ti OR hemiartroplasty:ti,ab) AND (proximal humerus fracture:ti,ab OR proximal humeral fracture:ti,ab OR shoulder fracture:ti,ab)
CENTRAL (n=63)<br>CINAHL (n=41)	(AB (conservative treatment OR non operative treatment OR sling) AND AB (reverse shoulder arthroplasty OR shoulder arthroplasty OR total shoulder replacement) AND AB (proximal humerus fracture OR shoulder fracture OR proximal humeral fracture))

Supplementary Material Table S2. Quality assessment according to the MINORS criteria.

Criteria	Reported and adequate (2)	Reported but inadequate (1)	Not reported (0)
Clearly stated aim	Aim including outcomes reported	Aim reported without outcomes	Not reported
Inclusion consecutive patients	Inclusion/exclusion criteria reported	Unclear description inclusion/exclusion criteria	Not reported
Prospective collection data	Prospective	Not applicable	Not applicable
Appropriate endpoints	Appropriate endpoints to aim study	Endpoints not appropriate to aim study	Not reported
Unbiased assessment	Blinded evaluation of outcomes	Reason not blinding stated	Not reported
Appropriate follow-up	≥ 1 year	< 1 year	Not reported
Loss to follow-up < 5%	≤ 5%	> 5%	Not applicable
Prospective calculation study size	Prospective power-analysis performed	Prospective calculation without power-analysis	Not applicable
Adequate control group	ORIF versus MIPO treatment	Not applicable	Not applicable
Contemporary groups	Study/control group managed during same period	Study/control not managed during same period	Not reported
Baseline equivalence groups	Baseline characteristics described and comparable	Baseline characteristics not comparable	Not reported
Adequate statistical analyses	Statistical analysis described including type of analyses	Inadequate description statistical analysis	Not reported

Items are scored 0 (not reported), 1 (reported but inadequate) or 2 (reported and adequate). The overall score ranging from 0 to 24 for comparative studies

Supplementary Material Table S3. Baseline characteristics of non-comparative studies and case series on RSA

Author	Country	Period	Design	Mean follow up	Mean age	Gender	Fracture type		Implant	Approach	Cemented	Tuberosity treatment
		Year-year		Months	Years	% female	Included fracture types	Specified %			Yes/ No or % cemented	% reattached
Klein et al	Germany	02-04	Pros	33	74.9	70	3 & 4 part	25/75	Delta	AL	yes	0%
Cazeneuve et al	France	93-09	Retro	79	75	94	3 & 4 part	NR	Delta	AL	yes	0%
Young et al	New Zealand	03-05	Retro	22	77	100	3 & 4 part	20/80	SMR	DP	no	90%
Lenarz et al	USA	05-08	Retro	23	77	90	3 & 4 part	3/97	Aeq	DP	yes	100%
Valenti et al	France	04-08	Retro	23	78	93	3 & 4 part	7/20/73	Arrow	SL	yes	60%
Garrigues et al	USA	03-11	Retro	43	80	NR	3 & 4 part	13/87	Delta/ Aeq	SL & DP	yes	100%
Boyle et al	New Zealand	99-10	Retro	42	79.6	93	NR	NR	SMR/ Delta	DP	NR	NR
Grassi et al	Italy	9-12	Retro	22	75	100	3 & 4 part	53/47	Delta	DP	yes	100%
Forcada et al	Spain	09-11	Pros	29	74.7	87	3 & 4 part	16/84	SMR	DP	no	100%
Lopiz et al	Spain	09-12	Retro	33	81.7	81	3 & 4 part	14/43 ^	Delta	DP	yes	100%
Wolfensperger et al	Switserland	12-14	Retro	12	80	85	3 & 4 part	45/55	Aeq	DP	yes	100%
Cuff et al	USA	07-10	RCT	30	74.4	NR	head split. 3 & 4 part	NR	DJO	DP	NR	100%
vdMerwe et al	New Zealand	99-14	Retro	60	78.2	90	NR	NR	SMR/ Delta	DP	NR	NR
Klug et al	Germany	11-17	Retro	38	73.9	83	head split. 3 & 4 part	40/3/57	Delta	DP	yes	100%
Fraser et al	USA	13-17	RCT	24	75.7	92	3 & 4 part	40/60	Delta	DP	yes	100%
Schmalzl et al	Germany	14-16	Retro	22	76	86	3 & 4 part	31/69	Univers	DP	73%	100%
Simovitch et al	USA	09-13	Retro	34	77	69	3 & 4 part	NR	Exa	DP	yes	75%
Boileau et al	France	08-11	Retro	37	80	92	3 & 4 part	16/84	Aeq	AL & DP	yes	100%
Ohl et al	France	10-15	Retro	28	77.7	87	3 & 4 part	NR	NR	AL & DP	88%	71%
Torrens et al	Spain	10-12	Retro	29	77.9	76	3 & 4 part	17/83	Delta	AS	yes	100%

Barbosa et al	Portugal	13-17	Retro	38	73.5	94	3 & 4 part	29/71	NR	DP	yes	100%
Pierfrancesco et al	Italy	09-14	Retro	63	77	87	3 & 4 part	29/71	SMR	DP	NR	82.00%
Wright et al	USA	08-15	Pros	32	71	87	3 & 4 part	4/23/73	Zimmer	DP	No	100%
Grobenhofer et al	Switserland	05-13	Retro	35	77	88	head split. 3 & 4 part	20/8/75	Zimmer	NR	NR	92%
Bonnevialle et al	France	09-11	Retro	39	78	90	4 part	100	NR	DP & AL	NR	100%
Repetto et al	italy	NR	Retro	42	71.2	NR	head split. 3 & 4 part	NR	SMR	DP	yes	NR
Baudi et al	Italy	08-12	Retro	27	77	NR	3 & 4 part	28/72	Zimmer/ Delta	NR	NR	NR
Solomon et al	USA	07-11	Retro	43	77	81	3 & 4 part	NR	Aeq	DP	yes	100%
Defzuli et al	USA	06-14	Retro	34	78	85	NR	NR	Exa	DP	yes	NR

NR: not reported
Pros: prospective. Retro: retrospective. RCT: randomized control trial
^: rest were fracture dislocation. unknown Neer classification
Delta: Delta III (DePuy Orthopaedics)
SMR: Modular Shoulder System (Lima-LTO)
Aeq: Aequalis Reverse-Fracture prosthesis (Tornier)
Arrow: ARROW universal shoulder prosthesis (Fh-Orthopaedics)
DJO: Reverse Shoulder Prosthesis (DJO Surgical)
Univers: Revers total shoulder system (artrex)
Exa: Equinoxe Reverse System (Exactech)
Zimmer: Reverse Anatomical Shoulder System (Zimmer)

Supplementary Material Table S4. Functional outcome scores and weighted mean scores of case series and non-comparative studies on RSA

Author	N cases	CMS	SD or Range	DASH	SD or Range	ASES	SD or Range	OSS	SD or Range	SST	SD or Range	PENN	SD or Range
Klein et al	20	67.9	14	46.9	16	68.0	12						
Cazeneuve et al	36	53.0	20-80										
Young et al	10					65.0	40-88	29.0	15-56				
Lenarz et al	30					78.0	36-98						
Valenti et al	27	55.0	44-71										
Garrigues et al	11					81.1	75-88					81.5	73-99
Boyle et al	55							41.5	2				
Grassi et al	19	45.7	19-69										
Forcada et al	31	56.1	24-80	17.5	12-30								
Lopiz et al	42	47.1	20-83										
Wolfensperger et al	33	71.0	12	30.0	21								
Cuff et al	24					77.0	67-82			7.4	6-9		
vdMerwe et al	218							37.6	4				
Klug et al	30	69.9	26	25.3	20	74.6	22	37.7	10				
Fraser et al	64	68.0	64-72*					40.8	39-43*				
Schmalzl et al	64	59.0	20-90			72.0	18-98						

Simovitch et al	55	64.1	11			78.3	16			8.8	3		
Boileau et al	38	64.0	15										
Ohl et al	420	57.0	15										
Torrens et al	41	60.7	10										
Barbosa et al	33	64.4	38-85	6.8 ^	0-50	78.3	30-98						
Pierfrancesco et al	55	72.5	3.8	16.8	5								
Wright et al	30					82.0	14			6.9	2		
Grobenhofer et al	51	62.0	21-83										
Bonnevialle et al	41			28.0 ^	14								
Repetto et al	27	58.5	8.5	28.6	12.3					6.7	2		
Baudi et al	25	56.2	15	40.4	25	69.3	15						
Solomon et al	16					79.0	72-86*						
Defzuli et al	13	70.0	NR			82.0	NR			9.1	NR		
		CMS		DASH		ASES		OSS		SST			
Number of studies		20		9		13		5		5			
Number of patients total		1124		281		361		377		149			

Weighted average score		60.0	24.6	75.8	38.5	7.8	na
NR: not reported<br>CMS: Constant Murley Score<br>DASH: Disability of Arm Shoulder Hand score<br>ASES: American Shoulder and Elbow Surgeons shoulder score<br>OSS: Oxford Shoulder Score<br>SST: Simple Shoulder Test OSS:<br>PENN: Penn shoulder score<br>*: 95% confidence interval<br>^: quickdash instead of full DASH<br><br>For functional outcome when only subgroup data concerning tuberosity treatment vs re-section, tuberosity healing vs non healed, cemented versus non cemented or early vs delayed was available the best outcome group was used. For external and internal rota-tion the score with arm in 0 degree abduction was used. Weighted average outcome scores were calculated for the RSA outcomes and the NOT outcomes. Weighing was done by study population only.							

Supplementary Material Table S5. Range of motion and weighted mean scores of case series and non-comparative studies on RSA

Author	N cases	Anterior elevation		Abduction		External rotation		Internal rotation	
		Degrees	SD or range	Degrees	SD or range	Degrees	SD or range	Degrees or Level reached	SD or Range
Klein et al	20	123	33	113	38	25		Lumbar	
Cazeneuve et al	36		5-9		4-9	1	1-4	1	1-4
Young et al	10	115	45-140			48	10-90	Sacral	
Lenarz et al	30	139	90-180			27	0-45		
Valenti et al	27	112	85-150	97	80-160	13	0-40		
Garrigues et al	11	121	90-145			34	10-45		
Grassi et al		111	50-160						
Forcada et al	31	120	40-180	113	50-170	5	0-10	3	0-6
Lopiz et al	42	126	85-170	117	55-160	22	0-30	Lumbar	
Cuff et al	24	139	102-172			24	8-42	46% full	
vdMerwe et al	218								
Klug et al	30	133	45	118	55	39	25	79	15
Schmalzl et al	64	119	26			29	16	Lumbar	
Simovitch et al	55	130	33	111	30	36	20	3	1
Boileau et al	38	135	29			24	14	5	3
Ohl et al	420	115	30			17	23	4	2
Barbosa et al	33	123	70-160	109	70-140	38	0-70	41	5-70
Pierfrancesco et al	55	135	13	119	12.8	28	7		
Wright et al	30	130	31			31	18	Lumbar	
Grobenhofer et al	51	118	40-165	111	40-165	18	0-65	5	0-10
Bonnevialle et al	41	130	30			23	20	Sacral	
Repetto et al	27	125	45	110	32	20	11	Sacral	
Solomon et al	16	110	100-130			28	25-30		

Defzuli et al	13	119		103		27		Lumbar	
Number studies		22		11		22			
Amount patients		1087		384		1104			
Weighted average score		122		112		22		N.a	

Blank field: authors did not investigate or report
N.a: not applicable

For functional outcome when only subgroup data concerning tuberosity treatment vs re-section, tuberosity healing vs non healed, cemented versus non cemented or early vs delayed was available the best outcome group was used. For external and internal rota-tion the score with arm in 0 degree abduction was used. Weighted average outcome scores were calculated for the RSA outcomes and the NOT outcomes. Weighing was done by study population only.

Supplementary Material Table S6. Functional outcome scores and weighted mean scores of NOT cohorts from studies not comparing with RSA

Author	Country	Period	Design	NOT compared to	Mean follow up	Age Included (mean)	N	Fracture type included	CMS score	OSS	DASH
					Months						
Clement	UK	92-96	Pros	-	12	>65 (76.9)	89	3, 4 part	59	-	
Boons	NL	04-09	Pros	HA	12	>65 (79.9)	25	4 part	60	-	
Rangan	UK	08-12	Pros	ORIF	24	>65 (subgroup)	61	2, 3, 4 part	-	36	
Brouwer	NL	04-14	Retro	-	46	>65 (72)	59	3, 4 part			33
Zyto	Sweden	90-93	Pros	ORIF	12	NR (75)	15	3, 4 part	65		
								Number of studies	3	1	1
								Number patients	129	61	59
								Weigted score	59.9	36	33

N: Number
CMS: Constant Murley Score
OSS: Oxford Shoulder Score
DASH: Disability of Arm Shoulder and Hand questionnaire
NR: not reported
Pros: prospective. Retro: retrospective. RCT: randomized control trial
UK: United Kingdom
NL: Netherlands
